# TOP‐LINE CSF Biomarker Outcomes from the Phase 2 Clinical Trial SHINE in Alzheimer’s Patients

**DOI:** 10.1002/alz.095767

**Published:** 2025-01-09

**Authors:** Valentina Di Caro, Eunah Cho, Nicole Knezovich, Kaj Blennow, Henrik Zetterberg, Charlotte Teunissen, Michael Grundman, Anthony O Caggiano, Mary E. Hamby

**Affiliations:** ^1^ Cognition Therapeutics, Inc, Pittsburgh, PA USA; ^2^ Cognition Therapeutics, Pittsburgh, PA USA; ^3^ Department of Psychiatry and Neurochemistry, Institute of Neuroscience and Physiology, The Sahlgrenska Academy, University of Gothenburg, Mölndal Sweden; ^4^ Institute of Neuroscience and Physiology, Sahlgrenska Academy at the University of Gothenburg, Gothenburg Sweden; ^5^ Neurochemistry Laboratory, Department of Laboratory Medicine, Amsterdam UMC, Vrije Universiteit Amsterdam, Amsterdam Neuroscience, Amsterdam Netherlands; ^6^ Global R&D Partners, LLC, La Jolla, CA USA; ^7^ Cognition Therapeutics, Purchase, NY USA

## Abstract

**Background:**

A Phase 2 randomized, double‐blind, placebo‐controlled 6‐month trial, SHINE (NCT03507790), was designed to include up to 144 participants with mild to moderate Alzheimer’s disease (AD) to study the effects of the sigma‐2 receptor (S2R) modulator CT1812 on cognition, safety, and biomarkers. CSF canonical and exploratory biomarkers were assessed to determine treatment effects with CT1812.

**Method:**

Participants (n = 153) received a daily oral dose of either CT1812 (100 mg or 300 mg) or placebo during the treatment period of 6‐months. The levels of CSF biomarkers Aβ40, Aβ42, pTau181, tTau, Nfl were measured by Lumipulse, neurogranin, GFAP and α‐synuclein by ELISA, SNAP‐25 and synaptotagmin by IP‐LC‐MS assay, and Aβ42/40 ratio calculated. An ANCOVA model was used to assess treatment effects comparing change from baseline values of CT1812 vs placebo. Significance was assessed at p<0.05 using 2‐tailed tests, and p‐values determined. A p‐value of <0.10 but >0.05 was considered evidence of a trend.

**Result:**

At the end of the 6‐month treatment period, change from baseline levels and assessment of treatment effects (p<0.05) for CSF Aβ40, Aβ42, pTau181, tTau, Nfl, neurogranin, GFAP, SNAP‐25, synaptotagmin, and αSynuclein will be presented for the full trial. In an interim analysis of the first 24 participants, a significant (p<0.05) change from placebo for Aβ40, Aβ42 was observed with 300 mg of CT1812, along with trends of changes in other biomarkers. Treatment effects for all biomarkers assessed in the entire study will be presented. We hypothesize that the effect seen in lowering Aβ40 and Aβ42 will persist in the full analysis.

**Conclusion:**

Understanding the effect of CT1812 on CSF biomarkers should lend support towards the clinical development of CT1812 for AD. The significant decrease in Aβ42and Ab40 levels may reflect pathway engagement given the mechanism of action of action of CT1812, and replication of this finding in the fully completed trial is prudent.

